# The Y chromosome and the heartache of males

**DOI:** 10.1186/2041-2223-3-9

**Published:** 2012-04-20

**Authors:** Antti Sajantila

**Affiliations:** 1 Department of Forensic Medicine, Laboratory of Forensic Biology, Hjelt Institute, SF–00014 Helsinki University, P.O. Box 40 (Kytösuontie 11), Helsinki, Finland

## 

It is well established that atherosclerotic coronary heart disease (CHD) presents sexual dimorphism in its incidence, age of onset, progression, treatment, morbidity and mortality. Men are more commonly affected and die more often and younger from CHD compared with their age-matched partners. Many algorithms, for example Framingham risk score, have been used to assess the risk of developing this disease with high incidence in the western world. The risk scores include overlapping parameters, such as age, sex, blood pressure, HDL cholesterol and smoking, but they also differ in some aspects. Yet, these factors do not provide prediction of risk for CHD similarly well for men or women.

In the March issue of *The Lancet*, Charchar *et al*. report a promising study of the role of the Y chromosome in coronary artery disease in men [1]. They studied 11 SNPs from the Y chromosomal phylogenetic tree in 3233 unrelated British men, who were subjects in three cardiological study cohorts. The genotype data showed that the men were descendants of nine Y chromosomal haplogroups (Hgs). For two of the cohorts, the authors analyzed associations between the Hgs and the risk of CHD. They found that 90% of the men belonged to two Hgs (R1b1b2 and I). This finding was more than interesting! The men with Hg I had an approximately 50% higher age-adjusted risk for CHD than men with other Hgs. Moreover, the association between Hg I and CHD was independent from other known biomedical and socioeconomic risk factors. In order to exclude population stratification or admixture as a bias to their study set, the authors used genome-wide SNP analysis for one of the cohorts. The subjects and controls were of European ancestry and no differences were observed between Hg I men and men of other Hgs. Being not completely satisfied with their finding of a new, independent risk factor for CHD, the authors continued to attempt to understand the mechanism behind this phenomenon. To tackle this question, Charchar *et al.* analysed the functional effect of the Y chromosome on the monocyte and macrophage transciptome in the third cohort. The authors showed that there are at least 19 molecular pathways that had differential expression between the Hg I men and the rest of the men with other Hgs. These 19 pathways were interconnected by genes related to immunity and inflammation. This observation also was interesting, since some of these genes are known to have a strong impact in development of atherosclerosis.

Why is this study of interest for the readers of *Investigative Genetics*, who do not necessarily follow journals such as *The Lancet* or medical studies in general? The answer is simple: many of the readers of *Investigative Genetics* apply the Y chromosome genotyping method used in this study to their own research and analysis interests. This study supports, that population and forensic geneticists are linked with their tools and studies and that an overlap may exist with medical studies. The study is a great example of translational medicine, an important topic in health today.
